# New and Novel Machine

**Published:** 1872-07

**Authors:** 


A NEW AND NOVEL MACHINE.—One of the most recent triumphs in mechanics is "Tilghman's Sand Blast;" a machine used for cutting or engraving glass and other hard substances ; and consists of a hopper filled with dry, sharp sand, which falls into a flexible tube, from which it is driven with great force by compressed air, upon the surface of glass to be cut. The sand instantly effaces the smooth surface of the glass, giving it a beautifully frosted appearance. By painting designs upon the glass, with ordinary paint, it is instantly frosted, save where the paint is placed, so that beautiful etchings and engravings are so obtained. The elasticity of the paint causes the sand to rebound, preventing it from cutting where the surface is so protected. It is said that perfect *fac similes* of the finest lace can be transferred to glass or metals without the slightest injury occuring to the lace. The instrument is being extensively employed in all manner of engraving, and its work surpasses that of the most skilled engraver.

